# Lifestyle intervention using the psychoeducational approach is associated with greater cardiometabolic benefits and retention of individuals with worse health status

**DOI:** 10.1590/2359-3997000000185

**Published:** 2016-08-23

**Authors:** Adriana Cezaretto, Camila Risso de Barros, Bianca de Almeida-Pititto, Antonela Siqueira-Catania, Milena Monfort-Pires, Luciana Gavilan Dias Folchetti, Sandra Roberta Gouvea Ferreira

**Affiliations:** 1 Departamento de Epidemiologia Faculdade de Saúde Pública Universidade de São Paulo São Paulo SP Brasil Departamento de Epidemiologia, Faculdade de Saúde Pública, Universidade de São Paulo (FSP-USP), São Paulo, SP, Brasil

**Keywords:** Diabetes prevention, depression, lifestyle, psychoeducation, retention

## Abstract

**Objective:**

This study aimed to compare the effects of two lifestyle intervention programs for type 2 diabetes mellitus (T2DM) prevention – traditional or interdisciplinary psychoeducation-based intervention – in daily habits and cardiometabolic risk factors and investigate the role of the psychoeducational approach for the retention of individuals in the program.

**Subjects and methods:**

Between 2008 and 2010, in a public health service, 183 pre-diabetic individuals were allocated to two 18-month interventions involving diet and physical activity. Physical activity, diet, quality of life (QOL) and depression and biochemical measurements were obtained. Linear mixed-effect models were used to assess the effect of the interventions. A student t test was used to compare dropouts versus non-dropouts.

**Results:**

Improvements in energy intake and physical activity were greater in the interdisciplinary than the traditional intervention. A decrease in fat mass and blood pressure was more pronounced with interdisciplinary intervention. Dropouts from the traditional intervention only had higher BMI and lower fiber intake and QOL than non-dropouts.

**Conclusion:**

The interdisciplinary psychoeducation-based intervention revealed useful for reducing cardiometabolic risk and retaining individuals with worse health profiles. This approach represents a feasible strategy for motivating high-risk individuals to adopt a long-term healthy lifestyle.

## INTRODUCTION

The modern lifestyle of populations, characterized by high consumption of energy-dense foods and physical inactivity, predisposes to weight gain and favors a growth in obesity-related diseases. In particular, abdominal obesity increases the risk of cardiometabolic disturbances, which in turn decrease the quality of life (QOL) and increase mortality ( 1 - 3 ).

Lifestyle interventions have yielded satisfactory results in preventing type 2 diabetes mellitus (T2DM) ( 4 - 6 ). In the Diabetes Prevention Program ( 5 ), modifications in dietary habits and physical activity were not only able to prevent T2DM but also induce greater improvement in QOL than the pharmacological arm, especially in individuals with considerable weight loss ( 7 , 8 ). However, long-term adherence to healthy behaviors remains a challenge for health professionals in real-life settings. This difficulty arises in part from the association of unhealthy habits with psychological problems. Among the latter, depression is highly prevalent and considered the number one cause of disability worldwide. This combination of unhealthy habits, excessive adiposity and mental disorders triggers a state of chronic low-grade inflammation and insulin resistance ( 9 , 10 ), which are underlying mechanisms of cardiometabolic diseases. Therefore, the management of psychological problems could facilitate achieving intervention goals and enhance QOL.

Psychoeducation is a therapeutic approach based on cognitive-behavioral theory, which involves the patients in solving their problems through communication and self-assertiveness training ( 11 ). Its effectiveness has been tested in prevention programs at the primary care level in developed countries ( 12 , 13 ) and was shown to be particularly effective when applied in interdisciplinary group sessions ( 14 ). Psychoeducational intervention was found to maintain benefits of psychiatric disease management with fewer relapses and higher levels of functioning ( 15 - 17 ) as well as improve the retention of alcohol-addicted individuals in a detoxification program ( 17 ). In clinical practice, the most disturbed patients and/or chronically ill, such as those with T2DM, are those who often fail to maintain treatment in healthcare systems.

In pre-diabetic conditions, psychoeducation was rarely applied in group sessions to promote a healthy lifestyle, being unclear whether this approach could improve retention of individuals at higher risk for T2DM.

Our group has reported benefits in QOL from lifestyle interventions in individuals at cardiometabolic risk treated under the Brazilian public health system ( 18 ). Psychoeducational techniques were applied in the interdisciplinary group sessions, whose participants showed greater improvement in QOL domains than those submitted to the traditional doctor-based intervention. A proportion of individuals interrupted participation in the interventions, which is worrisome since they are at risk for T2DM.

We hypothesized that an interdisciplinary psychoeducation-based intervention could be associated with greater clinical and psychological benefits and retention of individuals with a worse metabolic profile compared with a traditional intervention. Therefore, this study aimed to compare the effects of two lifestyle intervention programs for T2DM prevention – traditional or interdisciplinary psychoeducation-based intervention – in daily habits and cardiometabolic risk factors and investigate the role of the psychoeducational approach for the retention of individuals in the program.

## SUBJECTS AND METHODS

### Sample

A total of 438 individuals treated under the public health system in Sao Paulo, Brazil were screened for T2DM using a locally developed questionnaire ( 19 ) combined with a capillary to screen at-risk individuals. Those with values of random capillary glycemia > 140 mg/dL were invited to undergo clinical examination and laboratory tests. The inclusion criteria included the age range from 21 to 79 years and the presence of pre-diabetic conditions (impaired fasting glycemia and/or impaired glucose tolerance). Hypertensive participants had their blood pressure stabilized before starting the protocol, and anti-hypertensive schemes should be maintained during the intervention periods. The exclusion criteria included a medical history of neurological or psychiatric disturbances and liver, renal or infectious diseases. The institutional ethics committee approved the study and written consent was obtained from all participants. Those who developed diabetes were referred to medical treatment and pharmacological therapy. This trial was registered (RBR #65N292) on the ReBEC (ww.ensaiosclinicos.gov.br), the Brazilian registry center of the WHO International Clinical Trials Registry Platform.

**Figure 1:**
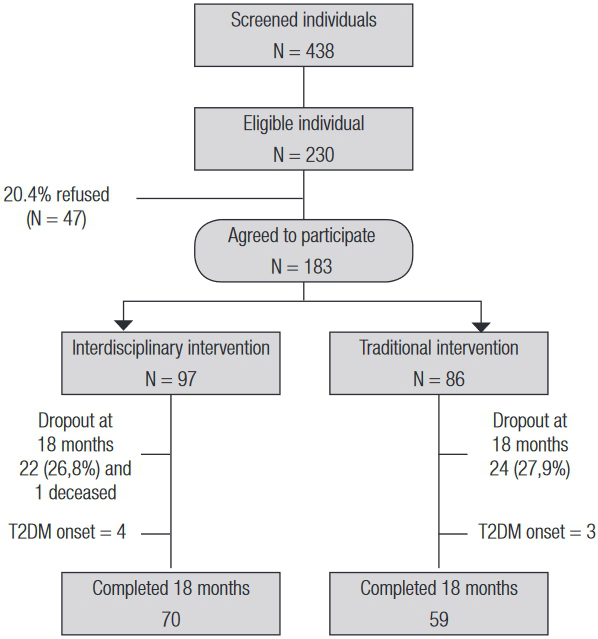
Flowchart of individuals in each stage of the study.

Of the 230 eligible, 183 individuals agreed to participate in the interventions (www.fsp.usp.br/prevsm). Those who refused to participate were predominantly men, whereas non-participants did not differ from participants in terms of baseline socio-demographic, anthropometric and metabolic variables. Of these 183 individuals, 129 completed the study ( Figure 1 ). The reasons for refusals were distance and time constraints to attend the intervention during business hours.

### Protocol

In this 18-month interventional study, individuals were randomly assigned to one of two programs of modifications in dietary habits, physical activity and stress management. They were examined at baseline and again after 9 and 18 months of follow-up.

The traditional intervention consisted of quarterly medical visits with an endocrinologist, in which participants received usual written guidelines for changing diet and physical activity advocated by the Brazilian public health system. In addition to the medical visits, the interdisciplinary intervention with the psychoeducational approach included an individual appointment with a nutritionist and 16 group sessions conducted by a multiprofessional team (endocrinologist, psychologist, nutritionist and physical educator). Four two-hour group sessions were provided in the first month, two sessions in the second, followed by seven monthly sessions until the ninth month and three quarterly sessions up to the 18^th^ month of the intervention (endpoint). Participants undergoing the interdisciplinary intervention also received printed materials to reinforce a healthy lifestyle. Group sessions dealt with issues related to daily routine, focusing on diet, physical activities and stressful situations and defining short-term targets. To enhance retention of the participants in the interventions, visits were arranged according to their convenience, and public transportation tickets were provided. Only for the interdisciplinary intervention, phone calls were made between sessions aimed at motivating and strengthening the participant-professional link.

Compliance was defined as 70% attendance to the interdisciplinary group sessions. Intervention goals were weight loss ≥ 5%, dietary fiber intake ≥ 20 grams/day, saturated fat ≤ 10% of total energy and moderate physical activity ≥ 150 minutes/week. The outcomes were the improvement in the risk profile, including blood pressure (BP) levels and glucose and lipid parameters.

### Measures and variables

Measures were obtained by trained professionals. The physical activity level was assessed by the long version of the International Physical Activity Questionnaire ( 20 , 21 ) and dietary habits by three 24-hour recalls (two weekdays and one weekend day). Dietary data were analyzed using the Nutrition Data System for Research software ( 22 ). The Beck Depression Inventory (BDI) was applied to detect depression symptoms; its scores ranged from 0 to 63, and a value > 11 is indicative of the presence of depression ( 23 ). QOL was assessed using the Medical Outcome Study 36-Item Short-Form Health Survey (SF-36). This questionnaire includes 8 QOL domains (physical function, physical role, bodily pain, general health, vitality, social functioning, emotional role and mental health). Combined scores allowed calculation of the SF-36 physical and mental component summary health scales ( 24 ). BDI and SF-36 were previously translated and validated for the Brazilian population, taking into account the degree of literacy ( 25 , 26 ).

Height was taken using a fixed stadiometer and weight with individuals wearing light clothes and no shoes on a Filizola digital scale. Body mass index (BMI) was calculated as weight divided by height in meters squared. Waist circumference was measured at the midpoint between the bottom of the rib cage and above the top of the iliac crest during minimal respiration. Fat mass was measured by bioelectrical impedance analysis using a Quantum II – BIA Analyzer (RJL Systems, Inc., Clinton Township, Michigan, USA). BP was measured in a sitting position, three times with a 5-minute interval using an automatic blood pressure device (Omron HEM-712C, Omron Health Care, USA). The average of the last two measurements was used in analyses.

Participants were submitted to a 75-gram oral glucose tolerance test. Plasma glucose and lipid profiles were immediately determined in the local laboratory using enzymatic methods. Aliquots were frozen at -80 ºC for further determinations. Insulin was determined by immunometric assay using a quantitative chemiluminescent kit (AutoDelfia, Perkin Elmer Life Sciences Inc, Norton, OH, USA) and adiponectin by ELISA (Human Adiponectin ELISA Kit, Millipore Corporation, MA, USA). Homeostasis model assessment (HOMA-IR) was used to assess insulin resistance ( 27 ).

### Statistical analysis

Physical activity, dietary intake, depression, QOL and clinical data were expressed as mean and standard error or deviation. A student *t* test (or the Mann-Whitney test when indicated) was used to compare baseline variables between genders and intervention groups as well as to compare individuals who dropped out from each intervention versus those who were retained. A chi-square test was employed to compare retention rates.

Generalized Linear Mixed Models (GLMMs) were used to examine the change differences of outcomes between the intervention groups, considering an unstructured covariance matrix, which has the advantage of analyzing repeated measures over time, even when incomplete. This method assumes the random effects following a normal distribution and that missing data are missing at random. Mixed-effect models were built for each dependent variable (lifestyle, anthropometric and biochemical variables and QOL), considering the interaction between time and interdisciplinary intervention at 9 or 18 months of follow-up. All models were adjusted for sex and age as well as for the interdisciplinary intervention in order to control for differences between interventions at baseline.

Statistical analyses were performed using SPSS version 17.0 for Windows (SPSS Inc., Chicago, Illinois, USA) and the R statistical package (R Foundation for Statistical Computing, Vienna, Austria). A p-value < 0.05 was considered significant.

## RESULTS

In the sample of 183 participants, 65% were women and the mean age was 54.7 ± 12.3 years. At baseline, 86% had a BMI ≥ 25 kg/m^2^ and 61% had pre-diabetes. Stratifying according to sex, women had significantly higher BMI (31.7 versus 29.1, p = 0.011), HDL-cholesterol (44.7 versus 37.4, p < 0.001), adiponectin concentrations (16.7 versus 10.8, p < 0.001) and depression scores (14.8 versus 6.5, p < 0.001) as well as lower QOL in both physical (47.0 versus 51.8, p < 0.001) and mental (41.9 versus 49.4, p < 0.001) components than men, respectively. Seven participants (three in the traditional and four in the interdisciplinary intervention) became diabetic during the period and were excluded from this analysis.

At baseline, participants allocated to the interdisciplinary intervention and had a worse clinical profile (higher mean values of anthropometric measurements and diastolic BP and lower adiponectin concentration) than those allocated to the traditional intervention ( Table 1 ).

**Table 1:** Baseline data according to type of intervention. Data expressed as mean (standard deviations) or percentages

	Traditional N = 86	Interdisciplinary N = 97	p-value
Women (%)	65.1	67.0	0.876
Schooling ≤ 8 yrs (%)	53.0	51.5	0.844
Employed (%)	65.0	59.8	0.106
QOL mental summary	44.7 (13.0)	44.2 (12.3)	0.807
QOL physical summary	49.1 (9.1)	48.3 (8.8)	0.532
Depression score^#^	11.4 (9.9)	12.4 (10.0)	0.441
Total energy intake (kcal)	1,754 (655)	1,861 (766)	0.315
Saturated fat intake (% total energy)	9.5 (2.6)	10.1(2.9)	0.188
Total fiber intake (per 1000 kcal)	9.2 (3.9)	9.1 (4.3)	0.883
Leisure physical activity (min/week)^#^	44.6 (73.4)	31.6 (61.7)	0.440
Body mass index (kg/m^2^)	29.9 (5.7)	31.7 (5.7)	0.041
Waist circumference (cm)	98.6 (13.4)	103.6 (11.9)	0.010
Fat mass (%)	32.9 (9.4)	35.6 (9.0)	0.053
Systolic blood pressure (mmHg)	134.3 (17.7)	137.6 (20.3)	0.240
Diastolic blood pressure (mmHg)	80.3 (10.0)	84.9 (11.2)	0.034
Fasting plasma glucose (mg/dL)	99.1 (10.9)	99.4 (12.1)	0.837
Post-load plasma glucose (mg/dL)	115.1 (26.9)	121.2 (27.6)	0.131
LDL-cholesterol (mg/dL)	124.3 (41.9)	127.6 (35.6)	0.561
HDL-cholesterol (mg/dL)	41.7 (11.7)	42.7 (11.8)	0.553
Triglycerides (mg/dL)	150.4 (71.5)	151.4 (66.5)	0.916
HOMA-IR^#^	2.4 (1.8)	2.6 (1.7)	0.460
Adiponectin (ng/mL)^#^	18.1 (17.6)	11.6 (7.2)	0.001

^#^ Mann-Whitney test used for comparisons. QOL: quality of life.

Interdisciplinary intervention induced lower energy intake along 18 months. Both groups exhibited similar patterns of change concerning saturated fat intake and fiber intake during the follow-up. However, participants of the interdisciplinary intervention had greater increase in leisure physical activity levels than those of the traditional one at the end of the follow-up ( Table 2 ).

**Table 2:** Adjusted models of changes after 9 and 18 months in each clinical and psychological measure (outcomes) considering interaction between time and interdisciplinary intervention, by linear mixed models*

	Coefficient (SE)	P-value
**Total energy intake (kcal)**		
Change at 9 months	-191.8 (56.7)	< 0.001
Change at 18 months	-131.1 (58.1)	0.025
Interaction time 9 mo X intervention	-137.6 (78.1)	0.079
Interaction time 18 mo X intervention	-268.4 (80.2)	< 0.001
**Physical activity (min/week)**		
Change at 9 months	28.1 (11.1)	0.012
Change at 18 months	-9.07 (11.7)	0.439
Interaction time 9 mo X intervention	1.69 (15.3)	0.912
Interaction time 18 mo X intervention	35.9 (16.1)	0.026
**Fat mass (percentage)**		
Change at 9 months	0.71 (0.50)	0.161
Change at 18 months	0.31 (0.55)	0.574
Interaction time 9 mo X intervention	-1.56 (0.69)	0.026
Interaction time 18 mo X intervention	-1.65 (0.74)	0.027
**Systolic blood pressure (mmHg)**		
Change at 9 months	0.48 (2.11)	0.819
Change at 18 months	-1.98 (2.17)	0.362
Interaction time 9 mo X intervention	-6.62 (2.92)	0.024
Interaction time 18 mo X intervention	-8.78 (3.01)	0.004
**Diastolic blood pressure (mmHg)**		
Change at 9 months	-0.46 (1.17)	0.691
Change at 18 months	-1.43 (1.21)	0.234
Interaction time 9 mo X intervention	-5.72 (1.62)	< 0.001
Interaction time 18 mo X intervention	-6.06 (1.67)	< 0.001
**Fasting glicemia (mg/dL)**		
Change at 9 months	-1.89 (1.66)	0.256
Change at 18 months	-5.72 (1.71)	< 0.001
Interaction time 9 mo X intervention	-2.11 (2.29)	0.357
Interaction time 18 mo X intervention	4.98 (2.35)	0.040
**Adiponectin (ng/mL)**		
Change at 9 months	0.18 (1.43)	0.899
Change at 18 months	2.71 (1.48)	0.067
Interaction time 9 mo X intervention	4.96 (1.98)	0.013
Interaction time 18 mo X intervention	2.11 (2.04)	0.301

* All models were adjusted for gender and age.

Considering psychosocial measures, both physical (β = 3.91, p < 0.001) and mental (β = 5.69, p < 0.001) QOL components increased, and depression scores (β = -5.04, p < 0.001) had decreased after 18 months, but no difference was found when analyzing the interaction between time and intervention.

Greater reductions in BMI (β = -0.79, p < 0.001) and waist circumference (β = -1.66, p = 0.023) were observed after 9 months of the interdisciplinary intervention, but the difference between interventions lost statistical significance after 18 months. A greater decrease in fat mass in the interdisciplinary intervention was maintained until the end of the follow-up. Systolic and diastolic blood pressure reduced significantly over time, and these reductions were consistently greater in the interdisciplinary intervention than in the traditional intervention when considering interaction between time and intervention ( Table 2 ).

The interdisciplinary intervention induced greater decreases in fasting plasma glucose than the traditional one only after 9 months. LDL-cholesterol and triglyceride concentrations did not change over time while significant elevations in HDL-cholesterol were observed for both intervention groups. HOMA-IR values decreased after 18 months (β = -0.88, p < 0.001), but no difference was found between interventions. Only participants of the interdisciplinary intervention had adiponectin concentrations increased at the ninth month ( Table 2 ).

**Figure 2:**
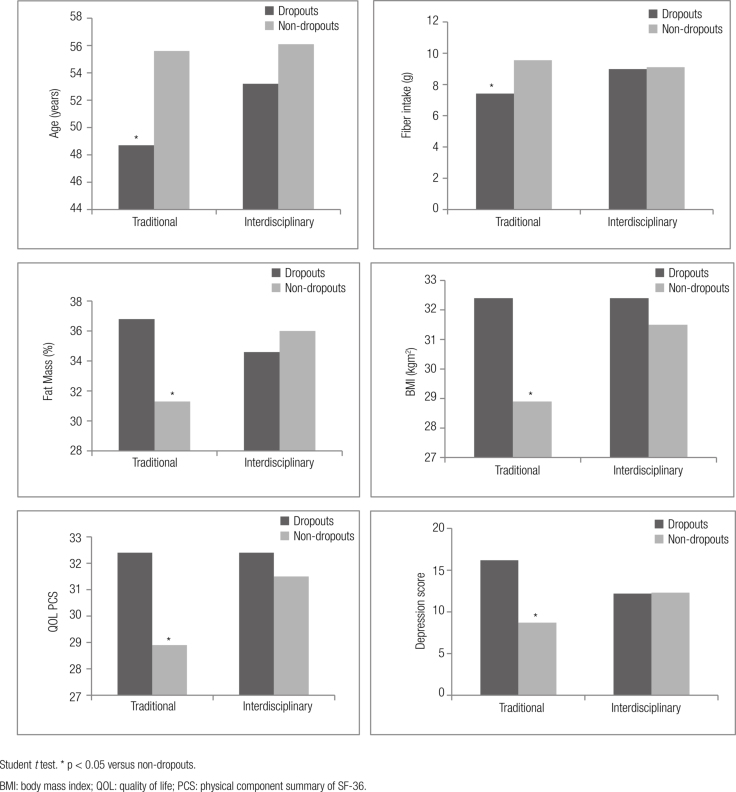
Mean values of baseline data for dropouts and non-dropouts according to type of intervention.

The percentage of individuals retained at the end of the follow-up was similar between interventions (traditional 70.7% and interdisciplinary 71.1%, p = 0.95). However, comparing baseline profiles, individuals who dropped out of the traditional intervention had worse depression scores, QOL, anthropometric measurements and dietary habits (lower fiber intake) than non-dropouts ( Figure 2 ). On the other hand, the lifestyle data and clinical profile of dropouts and non-dropouts from the interdisciplinary intervention did not differ.

Student t test. * p < 0.05 versus non-dropouts.

BMI: body mass index; QOL: quality of life; PCS: physical component summary of SF-36.

## DISCUSSION

Translating research evidence derived from lifestyle interventions into practice poses a challenge, particularly in populations of developing countries due to economic and social limitations. The strategies used to address the challenges identified in our intervention among Brazilians may facilitate the adoption of preventive programs in similar healthcare settings. The present study was able to demonstrate the superiority of an interdisciplinary intervention involving dietary and physical activity habits, which induced more pronounced benefits in body fatness, blood pressure levels and adiponectin concentrations than the traditional medical-centered intervention in individuals at cardiometabolic risk. Additionally, the psychoeducational approach retained individuals who most needed treatment, since this intervention was associated with greater retention of those exhibiting a worse metabolic profile at baseline.

By changing physical activity level and dietary habits, we sought to promote weight loss among the at-risk participants given the evidence of a protective effect against T2DM ( 4 - 6 ). In our study, the majority of the participants from the interdisciplinary intervention had increased time to do physical activity and decreased energy intake, suggesting that its approach could have encouraged this group to adopt healthier habits and resulting in favorable effects in body adiposity and blood pressure levels. During group sessions, participants had the opportunity to share problems and solutions by creating a forum for mutual help ( 28 ).

A beneficial effect of interdisciplinary intervention on anthropometric variables was seen at the ninth month; the rate of decline in fat mass but not in BMI was maintained until the end of the follow-up. The same pattern of changes, albeit to a smaller magnitude, was seen in the traditional intervention. This parallelism of the behaviors of both interventions could partially reflect a physiological defense against weight loss, mediated by a decrease in leptin levels ( 29 ). Although individuals from the interdisciplinary intervention started treatment with greater body adiposity than that of the participants from the traditional group, they showed more pronounced weight loss after follow-up. Previous studies have shown that motivational interviewing, such as psychoeducational groups, could be useful for achieving the goal of weight loss ( 30 , 31 ). The inclusion in our team of a professional who was aware of psychological concerns may have facilitated the maintenance of weight loss in the long term. This hypothesis is reinforced upon examining the clinical profile of individuals who completed each intervention.

The interdisciplinary intervention was able to retain a higher number of individuals with a worse clinical profile. By contrast, in the traditional intervention, individuals with lower fiber consumption, more severe obesity and worse QOL dropped out more frequently. These findings were interpreted as evidence that our psychoeducational approach improved motivation through life experience exchanges and mutual help. This probably helped participants manage their emotions, thereby facilitating behavioral changes. Healthier behavior and a better clinical profile were harder to be maintained by participants of the traditional group. Difficulties sustaining weight loss among obese individuals have been frequently reported, particularly in some specific populations such as low-income groups ( 32 , 33 ).

The benefits in cardiometabolic risk profile were achieved at the midpoint of our intervention but were attenuated by the end of the study. The same pattern has been described in other lifestyle interventions ( 34 ). Notably, we observed a sustained reduction in blood pressure for the interdisciplinary intervention only. This finding is in agreement with a 6-month trial adopting a similar approach to change lifestyle, in which significant blood pressure reduction was accompanied by weight loss ( 35 ). Such outcomes are relevant considering that even small decreases in these clinical parameters are able to lower the risk of chronic diseases such as T2DM, cardiovascular disease, osteoporosis and cancer ( 36 ).

Favorable effects in glucose and lipid metabolism were detected for both interventions. Plasma glucose and lipid concentrations decreased during the follow-up period, although no clear advantage of the interdisciplinary intervention was evident. Other behavioral population-based interventions have found clinical benefits, which were attributed to the mere fact that individuals had been followed up with ( 37 ). Unexpectedly, an increase in plasma glucose was detected after 18 months in the interdisciplinary group. Differences in the clinical characteristics of those retained in each intervention may have contributed to the more unfavorable outcomes of the participants in the interdisciplinary intervention. A relatively better metabolic profile of individuals from the traditional intervention at baseline might have also influenced the results. We speculate that this scenario may have attenuated the real benefits of the interdisciplinary intervention.

The limitations of our study include the small sample size and short duration of follow-up, precluding the evaluation of the incidence of concrete outcomes. Our reference group – the traditional intervention – is not representative of regular medical consultation under public healthcare systems since a specialist in endocrinology is not usually available in primary care units of developing countries. This could have reduced differences between the types of interventions, thus masking the real impact of the interdisciplinary intervention in its ability to change life habits. On the other hand, the results of the present study point to the importance of a traditional intervention having access to a diabetes specialist as an effective preventive strategy for high-risk individuals.

In conclusion, the interdisciplinary psychoeducation-based intervention proved useful for reducing cardiometabolic risk profile, most likely mediated by beneficial effects on daily habits. Moreover, this approach improves retention of at-risk individuals with worse health profiles. Interdisciplinary intervention, including group sessions, represents a feasible strategy for motivating individuals at a higher risk of adopting a long-term healthy lifestyle.
